# COVID-19 Vaccine Acceptance Rate and Its Factors among Healthcare Students: A Systematic Review with Meta-Analysis

**DOI:** 10.3390/vaccines10050806

**Published:** 2022-05-19

**Authors:** Muhammad Mainuddin Patwary, Mondira Bardhan, Md. Zahidul Haque, Rabeya Sultana, Md Ashraful Alam, Matthew H. E. M. Browning

**Affiliations:** 1 Environment and Sustainability Research Initiative, Khulna 9208, Bangladesh; mondirabardhan.22@gmail.com (M.B.); rtr.zahid@gmail.com (M.Z.H.); 2 Environmental Science Discipline, Life Science School, Khulna University, Khulna 9208, Bangladesh; rabeya.sultana@ku.ac.bd; 3 Department of Global Health Policy, Graduate School of Medicine, The University of Tokyo, Tokyo 113-0033, Japan; aalam@m.u-tokyo.ac.jp; 4 Tokyo Foundation for Policy Research, Tokyo 106-6234, Japan; 5 Department of Parks, Recreation and Tourism Management, Clemson University, Clemson, SC 29634, USA; mhb2@clemson.edu

**Keywords:** vaccine hesitancy, vaccine acceptance, COVID-19, frontline workers, healthcare students, meta-analysis, SARS-CoV-2, vaccine

## Abstract

Healthcare students are clinicians-in-training likely to come into contact with COVID-19 as much as other frontline healthcare professionals. It is therefore necessary to prioritize vaccinations for this group. We conducted a global systematic assessment of COVID-19 vaccine acceptance rates and related factors among healthcare students using the PubMed, Scopus, and Web of Science databases and keyword searches in March of 2022. We found 1779 articles with relevant information and 31 articles that matched our inclusion criteria. We performed a random-effects meta-analysis and quality assessment using the eight-item Joanna Briggs Institute Critical Appraisal test for cross-sectional studies. A total of 30,272 individuals from 16 countries were studied. Most of the studies were carried out in the U.S. (*n* = 6), China (*n* = 5), Poland (*n* = 5), India (*n* = 2), Italy (*n* = 2), and Israel (*n* = 2). The prevalence of the COVID-19 vaccine acceptance rate was 68.8% (95% confidence interval [CI]: 60.8–76.3, *I*^2^ = 100%), and the prevalence of the vaccine hesitancy rate was 25.8% (95% CI: 18.5–33.8, *I*^2^ = 99%). In country-specific analyses, Romania showed the highest acceptance rate (88.0%, 95% CI: 44.5–100%), while Iraq showed the lowest acceptance rate (66.2%, 95% CI: 35.5–90.8%). In time-trend analyses, we found that acceptance rates among healthcare students decreased over time. Students concerned about potentially serious side effects of the vaccine were less willing to accept the vaccine. National and international interventions should be adopted to reduce COVID-19 vaccination hesitancy rates among these important frontline workers.

## 1. Introduction

The ongoing COVID-19 pandemic has turned into a global challenge due to its dramatically contagious nature. The virus has led to more than 4.6 million deaths globally between February of 2020 and August of 2021 [1]. Vaccinations are one of the most cost-effective and long-lasting measures in helping to control such a public health disaster [2]. Vaccination rates directly impact herd immunity. Studies reported that when a population’s acquired immunity reaches 67%, the prevalence of COVID-19 infections will continue to decline [3]. Multiple biological and chemotherapeutic measures (i.e., plasma therapy, hydroxychloroquine, remdesivir, and tocilizumab) have been used to treat COVID-19 patients, but their curative effects have generally not been recommended or proven for patient treatment [4]. Many countries have encountered ongoing surges in COVID-19 cases due to relaxed precautionary measures like lockdowns, social distancing, hand washing, and personal hygiene practices [5]. Vaccinations remain the most important tool in combatting the pandemic.

Scientific authorities have undertaken over 300 vaccine invention projects, among which approximately 40 are in the clinical trial stage and a few are available to the general population [6]. Vaccine development was accelerated when two of these vaccines were granted Emergency Use Authorization (EUA) in December of 2020—a process that generally takes several years or more [7]. As of July of 2021, about three billion doses of COVID-19 vaccines have been administered worldwide. More than 11.48 billion doses of vaccines have been approved in at least one country. Clinical trials have shown that some of these have significant promise for real-world use, while others are customized to the unique needs of certain groups (i.e., older adults). Vaccination effectiveness generally varies between 50% and 77%. There is evidence that many vaccines may help minimize the likelihood of severe illness and asymptomatic disease, thus limiting the spread of SARS-CoV-2 [8,9].

Any vaccination program’s success depends upon people’s willingness to be vaccinated, the demand for vaccines, and positive attitudes toward vaccines [10,11]. Therefore, vaccine hesitancy limits the success of a vaccination program’s success; such hesitancy is defined by the indecision, reluctance, or refusal of vaccination [12,13]. The World Health Organization (WHO) has stated that vaccine hesitancy is a serious threat to public health [14]. For example, the 2018 measles outbreak in New York City revealed that vaccine hesitancy resulted in continuous transmission [15]. Vaccine hesitancy has been linked to numerous factors, such as distrust in the government, fear of side effects, and religious convictions [16].

During the COVID-19 pandemic, there has been much discussion about vaccines, especially among healthcare workers (HCWs) and students [17,18]. Healthcare students (HCSs) in medical, dental, nursing, and related programs are future clinical caregivers and important populations who need to be vaccinated against COVID-19. Several governments have chosen to incorporate medical students as volunteers who assist with coronavirus treatment while finishing their residency training [19]. HCSs are likely come into contact with COVID-19-infected patients during training sessions and clinical practice [20,21]. To avoid further infection and increase vaccine acceptance rates, medical students must be taught about the benefits of vaccines as part of their training. Furthermore, their families and friends look to them as competent and trustworthy resources of information, which means their opinions and views have an influence on the general public’s vaccine acceptance levels [22].

Numerous studies have investigated vaccine acceptance or hesitancy rates among HCSs. Some of these studies showed surprisingly high rates of vaccine hesitancy [17]. For example, one study in the U.S. found that nearly one-quarter of medical students were reluctant to be vaccinated, even after an approved COVID-19 vaccine was available [18]. Another study among nursing students in Greece, Albania, Cyprus, Spain, Italy, the Czech Republic, and Kosovo found that less than one-half were willing to be vaccinated [23]. In contrast, nearly 90% of medical students in India [24] and nursing students in China [2] were willing to get vaccinated.

We conducted a rapid systematic review with meta-analysis on HCSs’ perception of being vaccinated in response to this growing body of literature on vaccination acceptance and hesitancy among HCSs and the seemingly disparate results. Comparable systematic reviews and meta-analyses have been conducted among general populations [10,25,26] and healthcare workers [27,28], but not HCSs. Our aim was to assess the acceptance and hesitancy of COVID-19 vaccination rates among HCSs globally and identify the factors predicting vaccine acceptance. We expected the findings to help understand the challenges associated with vaccine hesitancy among HCSs, as well as inform strategies for overcoming these challenges.

## 2. Materials and Methods

We followed the Cochrane Rapid Review guidelines to conduct a rapid systematic review with a streamlined but robust approach. The criteria were searches in English and peer-reviewed studies. Similar approaches have been used to provide time-sensitive information that informs decision-making surrounding COVID-19 immunization programs [29].

### 2.1. Search Strategy

We systematically searched three databases (PubMed, Web of Science, and Scopus) using the PRISMA checklist (http://www.prisma-statement.org/) on 5 March 2022. We utilized the following Medical Subject Heading (MeSH) terms as well as text words (tw) for COVID-19: “COVID-19”, “SARS-CoV-2”, “coronavirus”, “novel coronavirus”, “nCoV”, “2019-ncov”, “SARS-2”, and “severe acute respiratory syndrome coronavirus 2”. For vaccines, we used: “vaccines”, “vaccination”, “COVID-19 vaccines”, “vaccina”, “vaccine uptake”, and “SARS-CoV-2 vaccine”. For acceptance/hesitancy, we used: “vaccine hesitancy”, “vaccine hesitance”, “vaccine acceptance”, “vaccine confidence”, “vaccine safety”, “vaccination attitudes”, “vaccine rejection”, and “vaccine willingness”. We did not specify the population terms to avoid excluding potentially important and relevant articles. Additional articles were identified using the references and citation lists of articles and reviews found in the keyword searches via forward and backward citation tracking in Google Scholar.

### 2.2. Study Selection

**Figure 1:**
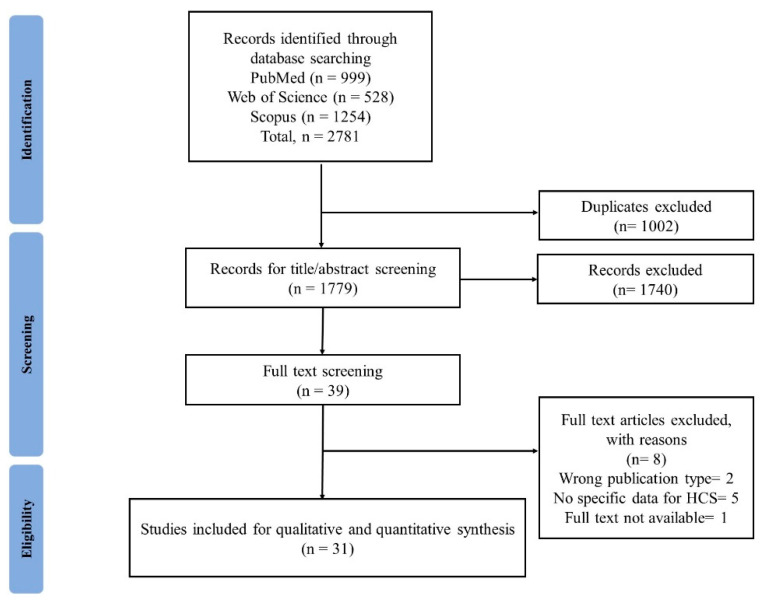
PRISMA flow diagram of the study selection process.

All records were imported to ‘Rayyan’ (https://www.rayyan.ai/; accessed on 5 March 2022). This is a tool for intelligent systematic reviews. Duplicates were removed using this software. Irrelevant records were excluded through title and abstract screening. Next, the full texts of the remaining articles were screened (Figure 1). Discrepancies were resolved by discussion among the three reviewers (MMP, MB, and MZH) and, if required, consultation with other co-authors for reaching a consensus. 

We had six inclusion criteria for the articles. These included: (1) survey studies among HCSs; (2) descriptive and observational studies among HCSs with cross-sectional, experimental, or longitudinal designs; (3) studies focused on evaluating COVID-19 vaccine acceptance and/or hesitancy; (4) studies published in English with no restriction to country; (5) studies published since January of 2020; and (6) peer-reviewed scientific journal articles.

Six exclusion criteria were included. These were: (1) articles not aiming to evaluate COVID-19 vaccine acceptance or hesitancy; (2) study populations other than HCSs; (3) publication types other than peer-reviewed journal articles, such as literature reviews, systematic reviews, unpublished data, books, conference papers, editorials, commentaries, letters to the editor, and case reports; (4) studies with non-human subjects; (5) studies without available full-texts; and (6) studies other than in English.

### 2.3. Data Extraction

Data extraction was performed independently by three co-authors. The extracted data included: author-name; publication year; study country; study design; survey method and period; target population; sampling method; sample size; measurement scale of vaccine acceptance; statistical analysis; acceptance rate; hesitancy rate; factors associated with vaccine acceptance, hesitance, or refusal; and summary of results. These data are summarized in Table 1. After independent data extraction, any differences were resolved by consensus among the same three co-authors.

### 2.4. Assessment of Study Quality

Regarding quality assessment and evaluating the risk of bias, we adopted the Joanna Briggs Institute critical appraisal tools for analytical cross-sectional studies (Table S1) [30]. This allowed us to determine whether certain articles should be included or excluded, or if additional information was required. We used a checklist with eight questions on the study’s methods and applicable data analysis for this purpose. The total score for each study was assessed by aggregating the individual scores and categorizing them into a high- or low-quality group following previous studies [31,32].

### 2.5. Data Analysis

Acceptance and hesitancy rates were pooled using random-effects models. The Higgin’s and Thompsons’s *I*^2^ statistics determined the heterogeneity [33,34]. Funnel plots and the Egger’s tests identified potential publication bias. We considered the survey year and country for subgroup analysis and conducted meta-regression analyses for four predictors: sex, residence, history of prior vaccinations, and concern about serious side effects. All analyses were performed using the ‘meta’ statistical packages in R software (version 4.2.1).

## 3. Results

### 3.1. Search Results

A total of 2781 articles were identified in preliminary searches across three databases including PubMed, Web of Science, and Scopus. Of these, 1002 articles were duplicates. After assessing their eligibility based on the title and abstract, 39 articles were eligible for full-text screening. Ultimately, 31 articles were included in the analyses (Figure 1).

### 3.2. Characteristics of Included Studies

The characteristics of the included articles are summarized in Table 1. Most used a cross-sectional design and collected data via telephone or online surveys. The majority also relied heavily on snowball sampling (i.e., via social media or email) and convenience sampling for recruitment. Studies were mostly conducted between March of 2020 and March of 2021.

The total number of healthcare students included in the studies was 30,272. Sample sizes ranged from 104 in Israel [35] to 6639 in one study across 22 countries [36]. Approximately 19,425 students (64% of total sample) were female. Most of the studies were conducted in the U.S. (*n* = 6), China (*n* = 5), Poland (*n* = 2), India (*n* = 2), Italy (*n* = 2), and Israel (*n* = 2). The largest share of HCSs were medical students, followed by nursing and dental students.

**Table 1:** Characteristics of included studies.

SL	Author	Study Country	Type of Healthcare Students	Study Design	Survey Method	Survey Period	Sampling Method	Sample Size, *N*	Gender, Female (%)	Vaccine Acceptance Rate (%)
1	Al Janabi et al. [37]	USA	Osteopathic medical	Cross-sectional	Online	October 2020	NR	197	57.9	45
2	Bălan et al. [38]	Romania	General Medicine, Dentistry, Pharmacy and Nursing and Midwifery	Cross-sectional	Online	12 January until 3 March 2021	NR	1581	74.5	88
3	Belingheri et al. [39]	Italy	Nursing	Cross-sectional	Online	21–27 December 2020	NR	422	82.9	80.9
4	Bolatov et al. [40]	Kazakhstan	Medical	Cross-sectional	Online	March 2021	NR	888	76.5	22.4
5	De Sousa Chaves et al. [41]	Brazil	Medical	Cross-sectional	Online	18 December 2020 to 8 January 2021	Snowball sampling	250	58.5	84
6	Gao et al. [42]	China	Medical	Cross-sectional	Online	February–March 2021	Convenience sampling	612	63.2	NR
7	Gotlib et al. [43]	Poland	Nursing undergraduate students	Cross-sectional	Online	March–April 2021	NR	793	90.8	38
8	Grochowska et al. [44]	Poland	Medical	Cross-sectional	Online/Off line	4 September–5 November 2020	NR	419	*n* = 331	70.7
9	Jain et al. [45]	India	Medical	Cross-sectional	Online	2 February–7 March 2021	Respondent-driven sampling strategy	1068	48.6	89.4
10	Jiang et al. [2]	China	Nursing	Cross-sectional	Online	February–April 2021	Convenience	1488	84.27	1256
11	Kanyike et al. [4]	Uganda	Medical	Cross-sectional	Online	15–21 March 2021	Convenience	600	37.2	224
12	Katz et al. [35]	Israel	Medical	Cross-sectional	Online	December 2020	NR	104	61.5	91.35
13	Kelekar et al. [17]	USA	Medical	Cross-sectional	Online	November–December 2019	NR	167	NR	126
			Dental					248		135
14	L. Jain et al. [46]	India	Healthcare student	Cross-sectional	Online	November 2020–January 2021	Snowball sampling	655	61.98	63.82
15	Li et al. [47]	China	Medical	Cross-sectional	Online	15 March–30 March 2021	NR	2196	81.7	1291
16	Lindner-Pawłowicz et al. [48]	Poland	Medical	Cross-sectional	Online	8–31 December 2020	NR	350	NR	76.9
17	Lucia et al. [18]	USA	Medical	Cross-sectional	Online	NR	NR	167	57	126
18	Lo Moro et al. [49]	Italy	Medical	Cross-sectional	Online	20 November 2020–2 February 2021	NR	838	63.5	93.3
19	Mahdi [50]	Iraq	Medical	Cross-sectional	Online	2021	NR	810	60.2	33.83
20	Manning et al. [21]	USA	Nursing	Cross-sectional	Online	10 August–14 September 2020	NR	1029	87.7	466
21	Mascarenhas et al. [20]	USA	Dental	Cross-sectional	Online	2020	NR	248	58	136
22	Mayan et al. [51]	USA	Medical	Cross-sectional	Online	9 February–15 March 2021	NR	1899	64.3	93.31
23	Mose et al. [52]	Ethiopia	Medical and health science	Cross-sectional	NR	1–30 March 2021	Simple random sampling	420	41.7	58.8
24	Petravic et al. [53]	Slovenia	Medical & Healthcare students	Cross-sectional	Online	December 2020	NR	624	79.49	Medical: 82, Healthcare: 51
25	Riad et al. [36]	22 countries	Dental	Cross-sectional	Online	6–28 February 2021	NR	6639	70.5	63.6
26	Rosental and Shmueli [54]	Israel	Medical and nursing	Cross-sectional	Online	27 August–28 September 2020	NR	628	66.6	Medical: 282Nursing: 234
27	Saied et al. [6]	Egypt	Medical	Cross-sectional	Online	8–15 January 2021	Convenience sampling	2133	NR	34.9
28	Szmyd et al. [55]	Poland	Medical	Cross-sectional	Online	22–25 December 2020	NR	687	64.77	632
29	Talarek et al. [56]	Poland	Medical	Cross-sectional	Online	March and April 2020	NR	411	68.4	94.6
30	Zhang et al. [57]	China	Healthcare students	Cross-sectional	Online	16–20 August 2021	NR	631	79.71	77.81
31	Zhou et al. [58]	China	Nursing	Cross-sectional	Online	4–20 January 2021	NR	1070	82.1	51.9

Notes: NR, Not Reported.

### 3.3. Prevalence of Vaccine Acceptance and Hesitancy

**Figure 2:**
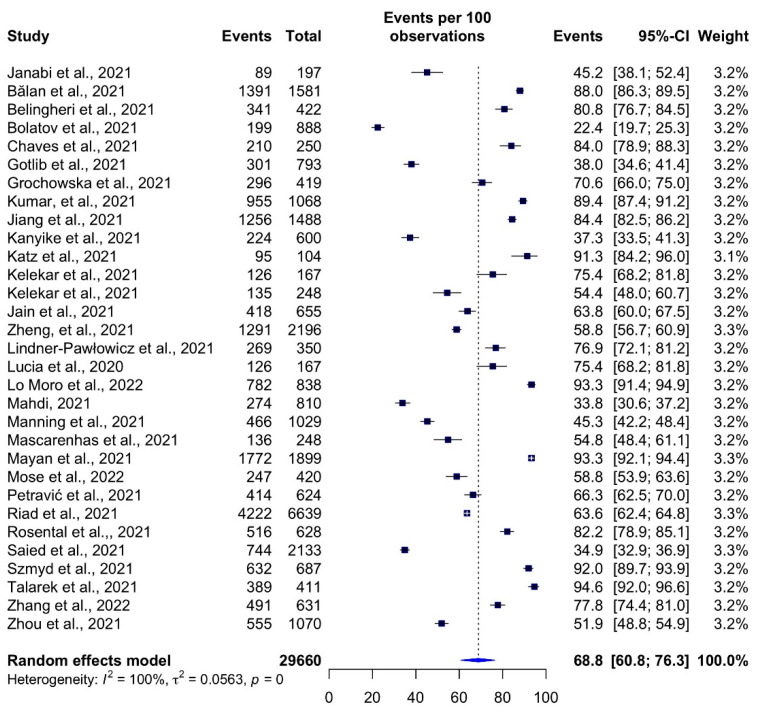
COVID-19 vaccine acceptance rates among healthcare students by study.

The estimated total COVID-19 vaccination acceptance rate among HCSs was 68.8% (95% CI: 60.8–76.3% *I*^2^ = 100%) (Figure 2). Talarek et al. [56] observed the highest acceptance rate (95.6%, 95% CI: 92.0–96.6%) in a study in Poland. The study in Kazakhstan by Bolatov et al. [40] reported the lowest vaccination acceptance rate of 22.4% (95% CI: 19.7–25.3%).

**Figure 3:**
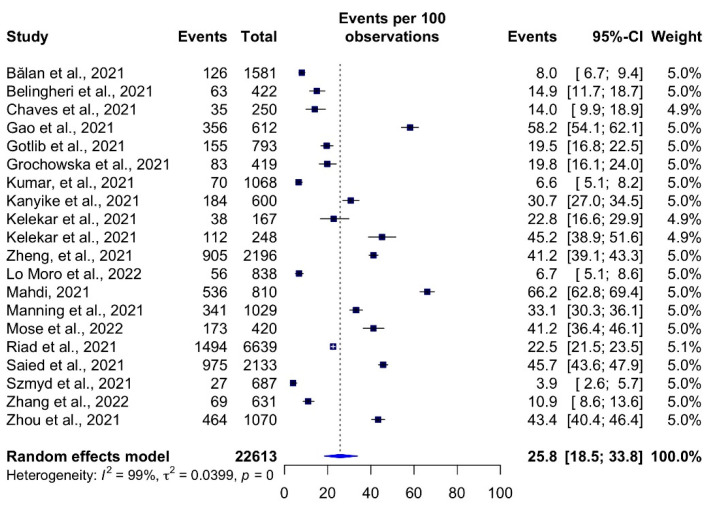
COVID-19 vaccine hesitancy rates among healthcare students by study.

The total estimated COVID-19 vaccination hesitancy rates among HCSs was 25.8% (95%CI: 18.5–33.8% *I^2^* = 99%) (Figure 3). Mahdi [50] reported the highest hesitancy rates in Iraq (66.2%, 95%CI: 62.8–69.4%), and the lowest rate of hesitancy was found in Poland (3.9%, 95% CI: 2.6–5.7%) by Szmyd et al. [55].

### 3.4. Sub-Group Analysis

**Figure 4:**
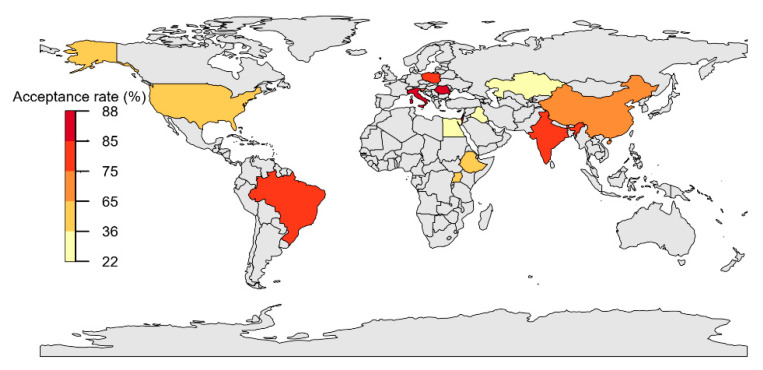
Map of COVID-19 vaccine acceptance rates among healthcare students by country.

**Figure 5:**
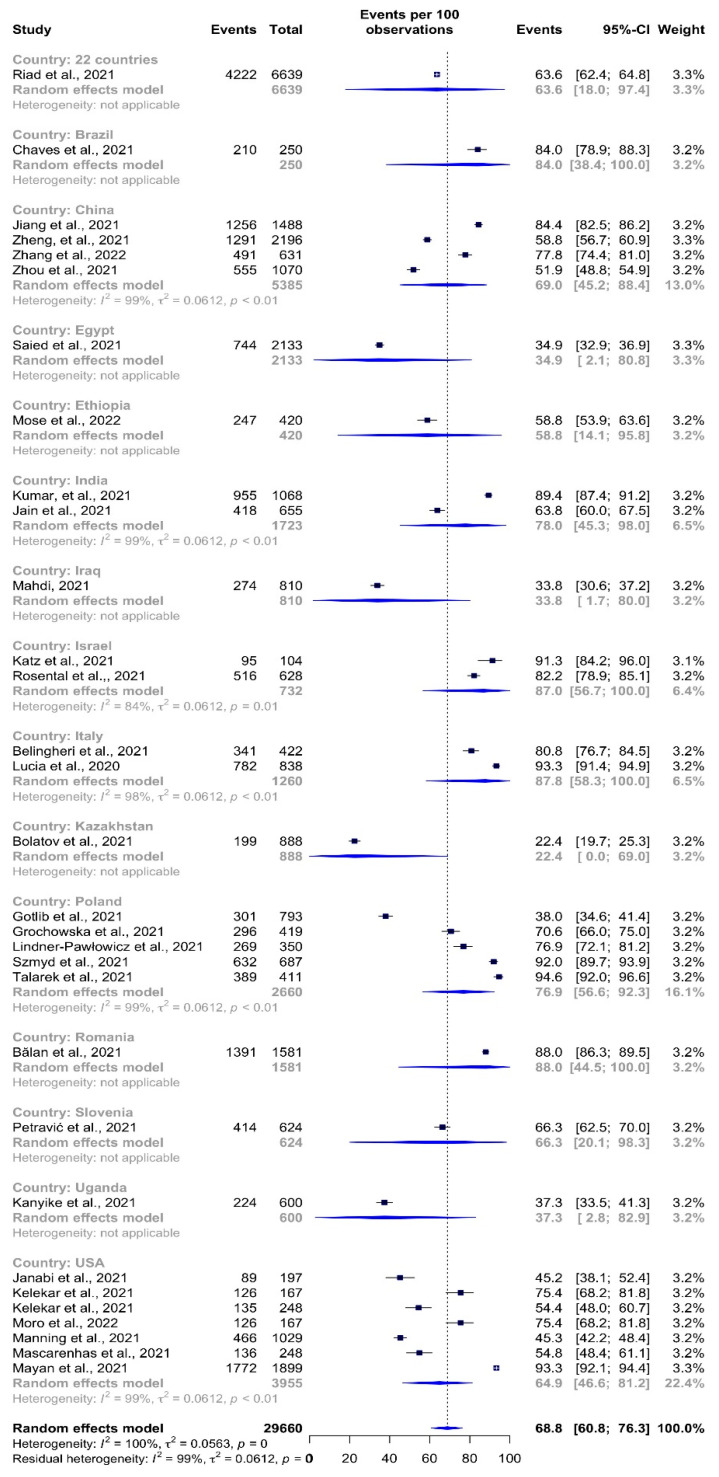
COVID-19 vaccine acceptance rates among healthcare students by country.

Figure 4 and Figure 5 present country-specific COVID-19 vaccine acceptance rates among HCSs. The pooled prevalence of the highest acceptance rate was observed in Romania (88.0%, 95% CI: 44.5–100%), followed by Italy (87.8%, 95% CI: 58.3–100%, *I*^2^ = 98%), Israel (87.0%, 95% CI: 58.7–100%, *I*^2^ = 84%), Brazil (84.0%, 95% CI: 38.4–100%), and India (78.0%, 95% CI: 45.3–98.0%, *I*^2^ = 99%).

**Figure 6:**
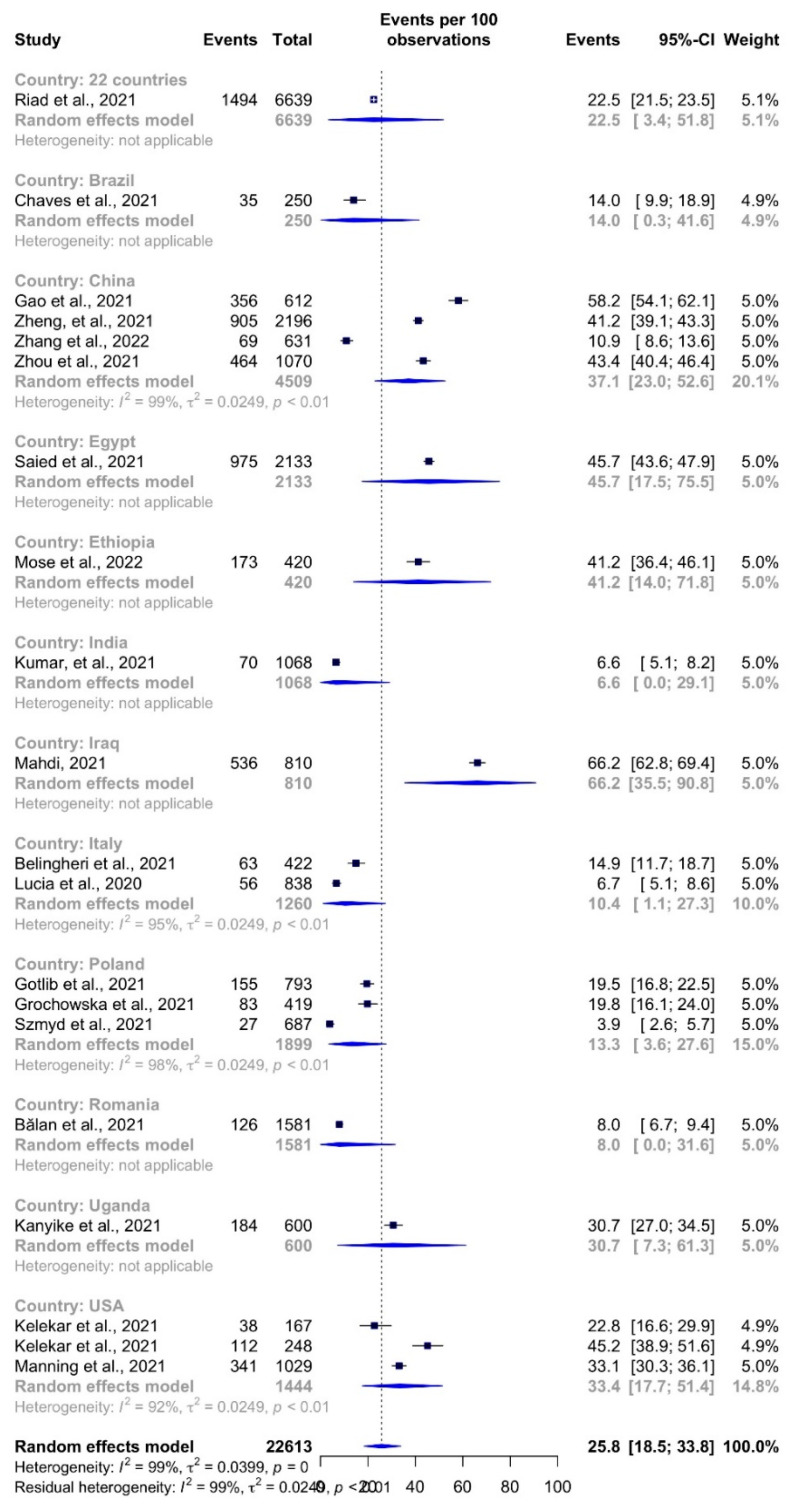
COVID-19 vaccine hesitancy rates among healthcare students by country.

Country sub-group analyses are presented in Figure 6. Iraq showed the highest rates of vaccine hesitancy (66.2%, 95% CI: 35.5–90.8%), followed by Egypt (45.5%, 95% CI: 17.5–75.5%), Ethiopia (41.2%, 95% CI: 14.0–71.8%), China (37.1%, 95% CI: 23.0–52.6%, *I*^2^ = 99%), and the U.S. (33.4%, 95% CI: 17.4–51.4%, *I^2^* = 92%).

### 3.5. Time Trends

**Figure 7:**
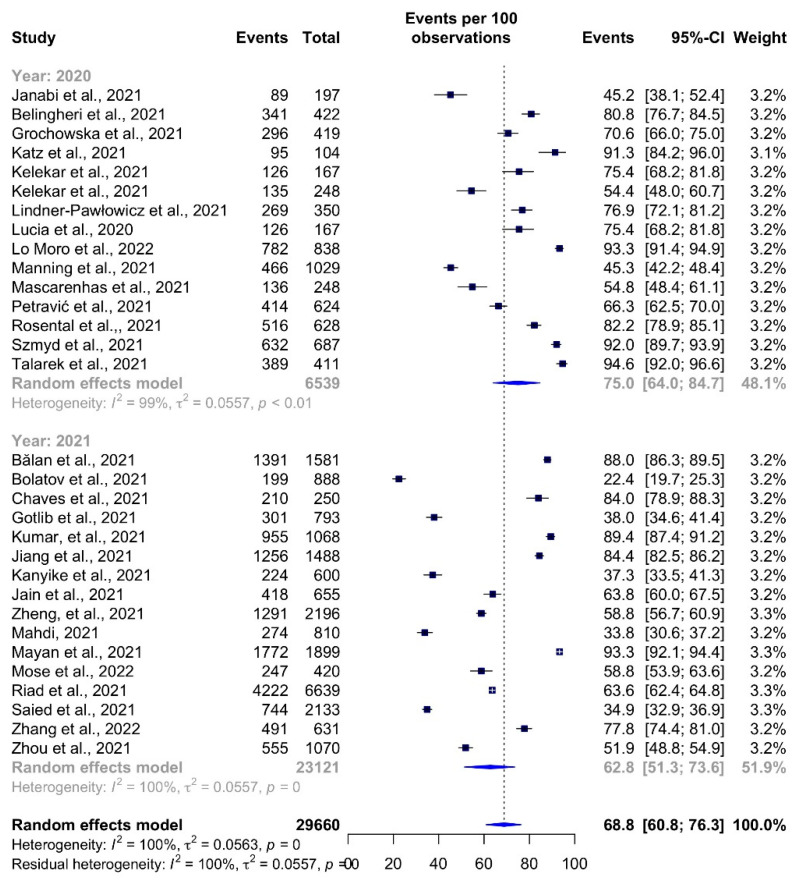
COVID-19 vaccine acceptance rates among healthcare students by year.

COVID-19 vaccine acceptance rates decreased with time (Figure 7). During 2020, the pooled acceptance rate was 75.0% (95% CI: 63.5–85.0%, *I^2^* = 99%). The acceptance rate in 2021 was only 62.8% (95% CI: 51.3–73.6%).

### 3.6. Predictors of Vaccine Acceptance

**Figure 8:**
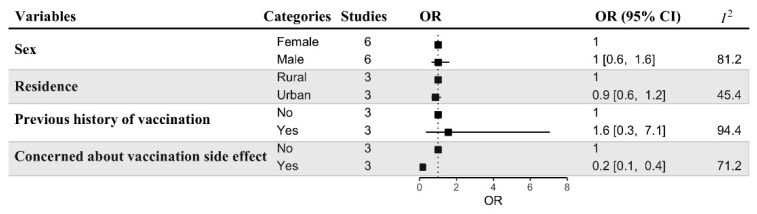
Predictors of COVID-19 vaccination acceptance among healthcare students.

Figure 8 presents the potential predictors associated with COVID-19 vaccine acceptance among HCSs. Sex, place of residence, previous history of vaccination, and concern about the vaccination side effects were considered. Only one factor—concern about potentially serious side effects of vaccines (*n* = 3 studies, OR = 0.2, 95% CI: 0.1–0.4)—was significantly associated with lower acceptance rates.

### 3.7. Risk of Bias

All 31 studies were assessed to be of the highest possible quality based on the JBI technique (Table S1). Studies that used ineffective recruitment methods like convenience and snowball sampling via social media were not removed, but their results may not have been representative of the population.

We observed no risk of publication bias. The Egger’s tests among studies of vaccine acceptance (*p*-value = 0.64) and vaccine hesitancy (*p*-value = 0.97) were not significant (Figures S1 and S2).

## 4. Discussion

### 4.1. Summary of the Main Findings

Vaccines have been revolutionary in their the potential to end the COVID-19 pandemic [38]. However, vaccine hesitancy remains high and an important obstacle in many vaccination programs [59,60]. Vaccine skepticism is on the rise among healthcare workers due in part to the rapid development of these vaccines [61]. Healthcare students can act as role models in their communities to increase trust about the safety of vaccinations [38]. Furthermore, healthcare students are frontline workers likely to be exposed to COVID-19 during training and clinical practice. It is necessary to prioritize vaccinations for this group. To our knowledge, no systematic review or meta-analysis had been conducted on vaccination acceptance and hesitancy rates among healthcare students.

The current study systematically reviewed and analyzed the data from 30,272 healthcare students across the world. Our pooled estimations showed that approximately two-thirds of healthcare students were willing to accept a COVID-19 vaccine. Meanwhile, approximately one-quarter were hesitant about accepting a COVID-19 vaccine. Such rates are similar to those observed in general populations [10] and healthcare workers [62]. One potential explanation for these findings is that healthcare students may be exposed to large amounts of health-related information, which may make them more aware of the vaccine’s serious side effects and thus influence their decision to be vaccinated [18].

Country-wise, pooled results found that healthcare students from comparatively high-income countries like Romania, Poland, Italy, and the U.S. were more likely to accept a COVID-19 vaccine than students in other countries. One possible explanation is that vaccines were more prevalent in higher-income countries, making it easier for students to receive vaccinations. A recent study reported that, among 25 countries, 10 high-income countries received a median of 51.7% more vaccine doses than their low-income counterparts (31–14.9%) despite high rates of authorization [63]. Furthermore, most of these studies were conducted during the early stages of the pandemic, when countries were experiencing increasing rates of COVID-19-related mortality. Fear of becoming infected could have influenced vaccine acceptance levels.

Low vaccine acceptance and high vaccine hesitancy were observed in Middle Eastern and African countries (e.g., Kazakhstan, Egypt, and Iraq). Middle Eastern results may be attributed to high belief rates in conspiracy theories and high dependence on social media platforms to obtain vaccine-related information [64]. Lower COVID-19 mortality rates might have influenced vaccine acceptance rates in African countries [65]. In addition, people in Africa have a history of vaccination skepticism, which may have contributed to low acceptance rates [66]. Traditionally, many African groups have shown poor health-seeking behaviors because of spiritual considerations that limit vaccination uptake [67].

Our study found that vaccine acceptance among healthcare students decreased over time. Earlier studies have also found that vaccine acceptance varies over time [68]. For example, a global systematic review on vaccine acceptancy rates reported a decline from 79% in March–May to 60% in June–October of 2020 [10]. This finding could be explained by the fact that students during the early stages of the pandemic were more fearful of being infected, which motivated them to receive a vaccine. Similar findings were observed among Egyptian medical students [6]. Additionally, Wong et al. [69] reported that individuals who were more fearful of COVID-19 demonstrated greater willingness to receive a vaccine due to the perceived benefit of immunization reducing the risk of infection. With time, healthcare students were exposed to more professional information, which likely influenced their decisions. Recent research shows that the observed decreases in vaccination intentions may be caused by COVID-19-related misinformation, as well as public worries about vaccine safety [70,71].

Finally, we found that concerns about serious side effects of COVID-19 predicted vaccine acceptance. A similar finding was observed in Egypt, where 74% of medical students reported that side effects were major barriers of vaccine acceptance [6]. Another study conducted among Egyptian healthcare professionals (HCWs) found that 57% of HCWs were unwilling to accept a vaccine due to their belief that vaccines were unsafe [72]. Such findings could be explained by students being doubtful of vaccine efficacy due to its rapid development. However, it is worth mentioning that different countries and regions often use different types of vaccines, and potential side effects vary, which may also influence vaccine hesitancy from study to study.

### 4.2. Implications

COVID-19 vaccinations should be prioritized for frontline workers since they are critical to COVID-19 responses and are at high risk of infection. Given the low degree of intention to vaccinate against COVID-19 among healthcare students, it is necessary to boost vaccine acceptability rates in this population. The majority of countries agree that frontline workers should be immunized against COVID-19 [27]. Our systematic review could be an initial step, as it estimated country-wise vaccine acceptancy and hesitancy coverage among healthcare students. This information could help decision-makers determine where and how to prioritize vaccine distribution. It is critical to focus on establishing confidence in COVID-19 vaccinations among this population. Governments of each country could mandate vaccination policies for not only healthcare workers but also healthcare students.

### 4.3. Strengths and Limitations

This study has a number of strengths. It is the first comprehensive meta-analysis study on vaccination acceptability among healthcare students that we are aware of. All of the publications considered in this review were judged as high-quality observational studies. Our evaluation considered the most recent study findings when calculating the final vaccination acceptance rate.

Our review also has limitations. First, our search was confined to three databases (Scopus, PubMed, and Web of Science). Other databases, such as Embase, PsycINFO, CINAHL, PMC, or NCBI were not searched. Secondly, we excluded preprints and unpublished grey literature. Given the spike in COVID-19 papers throughout our research period, we may have reached a different outcome if preprints or unpublished grey literature were included. Third, the data collection period for the included studies was from 2020 to early 2022, which may have influenced the findings due to the fact that public sentiments regarding vaccination change over time. Fourth, most of the reviewed research was cross-sectional and performed through online surveys. Conclusions from online research are prone to clarity and self-selection bias [73]. Finally, we were unable to investigate some potential determinants of vaccine acceptance owing to data constraints.

## 5. Conclusions

Healthcare students were moderately willing to accept a COVID-19 vaccine as of March of 2022. Romania and Kazakhstan showed the highest and lowest vaccine acceptance rates, respectively. Vaccination acceptance rates among healthcare students decreased from 2020 to 2021. Healthcare students who expressed concerns about the potential side effects of the vaccine were less likely to accept a vaccine.

Governments should prioritize vaccine distribution to frontline healthcare workers, including students, as soon as safe vaccines are available. These efforts should be coupled with comprehensive educational programs that reinforce the safety of vaccines to healthcare students. Previous studies indicate that vaccine-exposed medical students have positive attitudes toward vaccines. If more healthcare students are vaccinated, they can relate their positive experiences to their patients and increase vaccine uptake in the general public.

## Data Availability

Data generated in this study is available by contacting the first author, Muhammad Mainuddin Patwary, if requested reasonably.

## Supplementary Materials

The following supporting information can be downloaded at: https://www.mdpi.com/article/10.3390/vaccines10050806/s1, Figure S1: Funnel plot of vaccine acceptance among healthcare students. Figure S2: Funnel plot of vaccine hesitancy among healthcare students.; Table S1: Quality assessment of included studies.
