# Supplementary material for: COVID-19 Vaccine Acceptance Rate and Its Factors among Healthcare Students: A Systematic Review with Meta-Analysis
Source: Vaccines (Basel). 2022 May 19;10(5):806. doi: 10.3390/vaccines10050806 (PMC9143226; doi:10.3390/vaccines10050806)
Supplement: Supplementary file 1 [file vaccines-10-00806-s001.zip › vaccines-1698123-supplementary.pdf]

Table S1. Quality assessment of included studies.

SL	Author	Country	1. Were the Criteria for Inclusion in the Sample Clearly Defined?	2. Were the Study Subjects and the Setting Described in Detail?	3. Was the Exposure Measured in a Valid and Reliable Way?	4. Were Objective, Standard Criteria Used for Measurement of the Condition?	5. Were Confounding Factors Identified?	6. Were Strategies to Deal with Confounding Factors Stated?	7. Were the Outcomes Measured in a Valid and Reliable Way?	8. Was Appropriate Statistical Analysis Used?	Quality Level (High: ≥50%; Low: <50%)
1	Al Janabi et al. (Al Janabi et al., 2021)	USA	1	1	1	1	0	0	0	1	High
2	Bălan et al. (Bălan et al., 2021)	Romania	1	1	1	1	0	0	1	1	High
3	Belingheri et al. (Belingheri et al., 2021)	Ialy	1	1	1	1	1	0	1	1	High
4	Bolatov et al. (Bolatov et al., 2021)	Kazakh-stan	1	1	1	1	0	0	1	1	High
5	De Sousa Chaves et al. (De Sousa Chaves et al., 2021)	Brazil	1	1	0	1	0	0	1	1	High
6	Gao et al. (Gao et al., 2021)	China	1	1	1	1	0	0	1	1	High

7	Gotlib et al. (Gotlib et al., 2021)	Poland	1	1	0	1	0	0	1	1	High
8	Grochowska et al. (Grochowska et al., 2021)	Poland	1	1	0	1	0	0	1	0	High
9	Jain et al. (J. Jain, Saurabh, Kumar, et al., 2021)	India	1	1	1	1	0	0	0	1	High
10	Jiang et al. (Jiang et al., 2021)	China	1	1	1	1	0	0	0	1	High
11	Kanyike et al. (Kanyike et al., 2021)	Uganda	1	1	0	1	0	0	0	1	High
12	Katz et al. (Katz et al., 2021)	Israel	1	1	0	1	0	0	1	1	High
13	Kelekar et al. (Kelekar et al., 2021)	USA	1	1	1	1	0	0	1	1	High
14	(L. Jain et al., 2021)	India	1	1	0	1	0	0	1	1	High
15	Li et al. (Li, Zheng, et al., 2021)	China	1	1	0	1	0	0	1	1	High

16	Lindner-Pawłowicz et al. (Lindner-Pawłowicz et al., 2021)	Poland	1	1	0	1	0	0	0	1	High
17	Liucia et al. (Lucia et al., 2020)	Italy	1	1	0	1	0	0	0	1	High
18	Lo Moro et al. (Lo Moro et al., 2022)	USA	1	1	1	1	0	0	1	1	High
19	Mahdi (Mahdi, 2021)	Iraq	1	1	0	1	0	0	1	1	High
20	Manning et al. (Manning et al., 2021)	USA	1	1	0	1	0	0	0	1	High
21	Mascarenhas et al. (Mascarenhas et al., 2021)	USA	1	1	1	1	0	0	1	1	High
22	Mayan et al. (Mayan et al., 2021)	USA	1	1	1	1	0	0	0	1	High
23	Mose et al. (Mose et al., 2022)	Ethiopia	1	1	0	1	0	0	1	1	High

24	Petravic et al. (Petravić et al., 2021)	Slovenia	1	1	1	1	1	1	1	1	High
25	Riad et al. (Riad et al., 2021)	22 countries	1	1	1	1	0	0	1	1	High
26	Rosental and Shmueli (Rosental & Shmueli, 2021)	Israel	1	1	1	1	0	0	1	1	High
27	Saied et al. (Saied et al., 2021)	Egypt	1	1	0	1	0	0	0	1	High
28	Szmyd et al. (Szmyd et al., 2021)	Poland	1	1	0	1	0	0	0	1	High
29	Talarek et al. (Talarek et al., 2021)	Poland	1	1	1	0	0	0	0	1	High
30	Zhang et al. (Zhang et al., 2022)	China	1	1	0	1	0	0	1	1	High
31	Zhou et al. (Zhou et al., 2021)	China	1	1	1	1	0	0	1	1	High

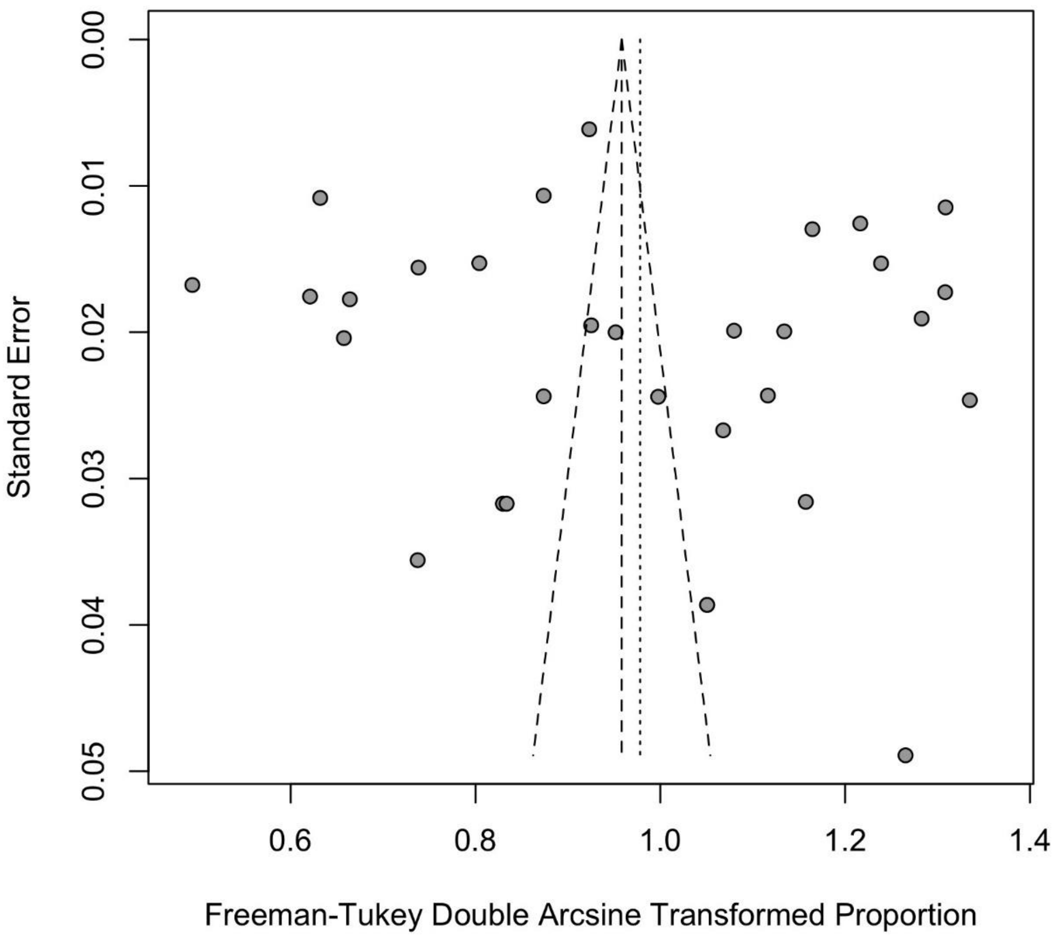

Figure S1. Funnel plot of vaccine acceptance among healthcare students.

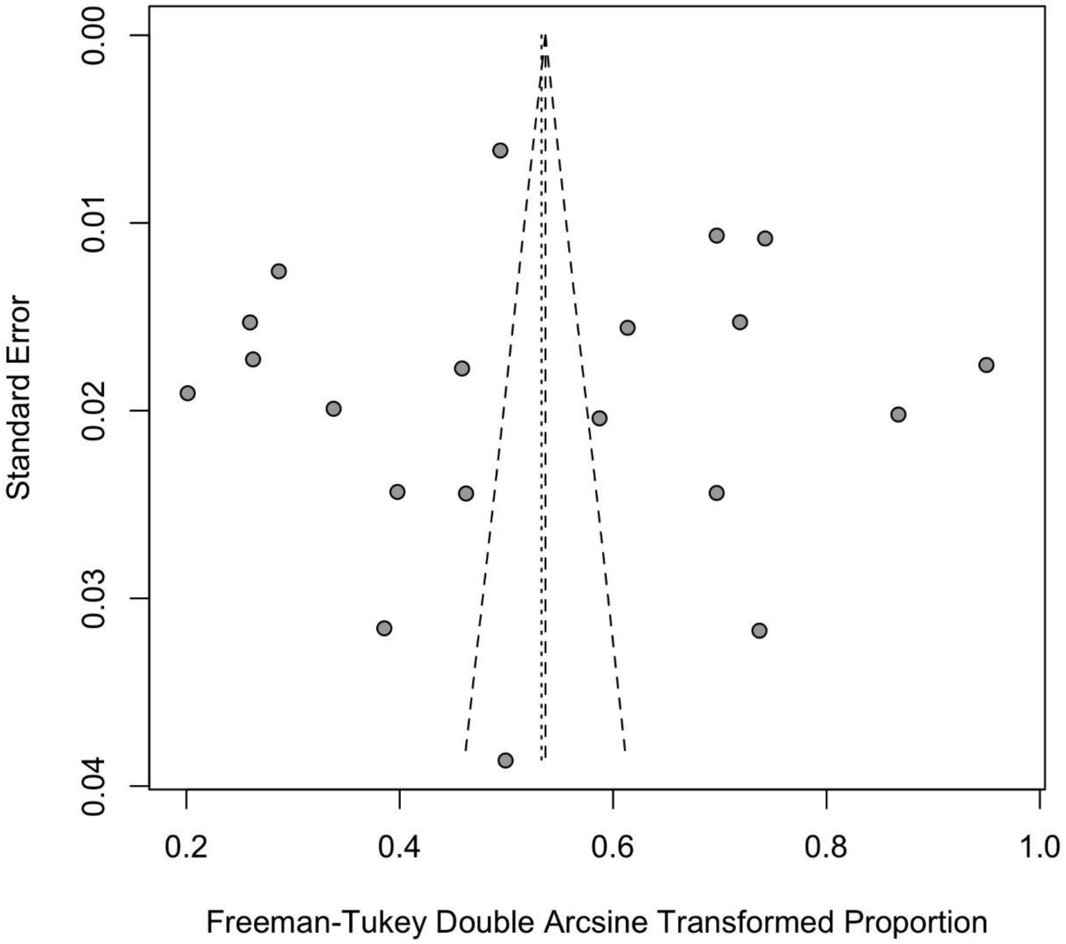

Figure S2. Funnel plot of vaccine hesitancy among healthcare students.
